# Bromide (Br) - Based Synthesis of Ag Nanocubes with High-Yield

**DOI:** 10.1038/srep10772

**Published:** 2015-06-09

**Authors:** Fan Wu, Wenhui Wang, Zhongfeng Xu, Fuli Li

**Affiliations:** 1 School of Science, Xi’an Jiaotong University, Xi’an 710049, China

## Abstract

The geometry of metal nanoparticles greatly affects the properties of the localized surface plasmon resonance and surface-enhanced Raman scattering. The synthesis of metal nanoparticles with controllable geometry has thus attracted extensive attentions. In this work, we report a modified polyol synthesis approach of silver (Ag) nanocubes through tuning the concentration of bromide ions (Br^−^ ions). We have systematically investigated the effect of Br^−^ ions in the polyol process, and find that higher concentration of Br^−^ ions can enhance oxidative etching effect, which is the dominative factor in determining nanostructure geometry. Therefore, one can realize control over nanostructure geometry by manipulating the concentration of Br^−^ ions. Our work provides an effective approach to control the shape of metallic nanostructures for potential applications.

With the development of nanoscience and nanotechnology, numerous kinds of nanomaterials are emerging and have been performing a variety of functions in different fields. Among these nanomaterials, metallic nanostructures are drawing particular interest due to their unique plasmonic properties, which endow them with promising applications in chemical and biological sensing^1^,^2^, energy harvesting^3^,^4^, waveguiding^5^,^6^, and nanophotonic circuits^7^,^8^. These applications are mainly determined by the localized surface plasmon resonance (LSPR), the frequency and intensity of which show strong dependence on the geometry (shape and size) of the nanoparticles^9^,^10^. Thus, the ability to control the shape and size of metal nanoparticles has become a crucial issue in the synthesis of metal nanostructures.

Currently there are two widely adopted methods: lithographic techniques^11^,^12^ and wet chemical methods^13^,^14^ to prepare nanostructures with uniform size and shape. Although the lithographic fabrication can control the shape of metal nanostructure, the lithographic techniques such as electron beam lithography, or focused ion beam lithography are complex, expensive, and require highly specialized facilities. In addition, the obtained nanostructures are almost always polycrystalline. In contrast, wet chemical synthesis method is more practical and economical in producing large quantities of nanostructures. In particular, the polyol process method developed by Xia’s group has successfully synthesized high-crystallinity metal nanostructures with various geometry, including metal spheres^15^, rods^16^, plates^17^, and wires^18^. Among these metal nanostructures, silver nanocubes have drawn increasing interest due to their unique properties such as plasmonic Fano resonance, which can greatly improve the sensitivity of LSPR based sensors^19^,^20^. Meanwhile, enhanced local electromagnetic fields at the 8 vertices of cube can enhance optical nonlinear process^21^. Therefore, the preparation of nanocubes with desirable geometry is essential. Additive ions such as chloride ions, sulfide ions, and ferrous ions have been adopted in the polyol process to prepare Ag nanocubes^22^,^23^,^24^.

During the preparation of Ag nanoparticles through using polyol process method, oxidative etching plays an important role in controlling the geometry and crystallinity of final products^25^. The effect of oxidative etching has been demonstrated in several systems with the ability to manipulate the ratio of twinned seeds to single-crystal seeds^23^,^25^,^26^. As final products are largely determined by the type of crystal seeds in the initial nucleation stage, the ability of oxidative etching effect to control the type of the crystal seeds is essential. The oxidative etching effect of Br^−^ ions has been adopted for the removal of multiple twinned crystal seeds in the preparation of Ag bipyramids^27^.

In this work, we report an effective approach for the preparation of monodisperse single-crystal Ag nanocubes using Br^−^ ions. As Ag nanocubes are supposed to originate from single-crystal seeds, multiple and single twinned crystal seeds in the initial nucleation stage need to be effectively eliminated. This can be achieved by enhanced oxidative etching effect of Br^−^ ions. We systematically investigate the influence of the concentration of Br^−^ ions on the geometry of Ag nanostructure, and find that oxidative etching effect increases with the increasing concentration of Br^−^ ions. Thus, we can realize control over oxidative etching effect through manipulating the concentration of Br^−^ ions. The preparation of Ag nanocubes under the effect of Br^−^ ions not only proves that we can obtain desired metal nanostructure by delicate control over the concentration of Br^−^ ions, but also provides strong evidence that high concentration of Br^−^ ions will enhance the oxidative etching effect.

## Materials and Methods

Two different volume ethylene glycol (EG, analytical grade) solutions, one 27 mL containing 94 mM silver nitrate (AgNO_3_, ≥99.8%), the other 135 mL containing 144 mM poly(vinyl pyrrolidone) (PVP, M.W. = 4,0000) and 0.144 mM sodium bromide (NaBr, ≥99.0%) were prepared. 3.2 mL of the two solutions were added dropwise in 0.375 mL/min via a two-channel syringe pump (LSP02-1B, Longer Pump) into 5 mL of EG heated in a one-necked round-bottomed flask in an oil bath at 160 °C under vigorous stirring conditions. The graham condenser was always used unless simultaneously injecting the PVP and AgNO_3_. A 30 μL drop of 13.6 mM sodium bromide (NaBr, ≥99.0%, EG as the solvent) was also added to the preheated EG before the Ag precursor. It’s worth noting that AgNO_3_ is not stable, easy to be decomposed when exposed to light. The preparation of the EG solution containing AgNO_3_ and the process of injection were all kept in a dark place. The final products were washed with acetone 1 time, ethyl alcohol 2 times, ultrapure water 1 time, respectively, and finally suspended in ethyl alcohol for future use.

The sample for transmission electron microscope (TEM) or scanning electron microscope (SEM) study was prepared by drying a drop of the aqueous suspension of particles on a piece of carbon-coated copper grid or silicon wafer under ambient conditions. TEM images were captured using a JEM-2100F transmission electron microscope operated at 200 kV, equipped with a SC200D lens-coupled CCD camera. SEM images were taken on a FEI field-emission microscope (JEOL JSM-7000F) operated at an accelerating voltage of 20 kV. The ultraviolet visible (UV) extinction spectrum was taken at room temperature on a Lambda 750S spectrometer (PerkinElmer). The solution had been diluted 6 times with ethyl alcohol before taking spectrum. The X-ray diffraction (XRD) pattern was recorded using a BRUKER D8 ADVANCE X-ray diffractometer with the Cu Kα radiation (λ = 1.54056 Å) at a scanning rate of 0.4 degrees per second in 2θ ranging from 30° to 80°. The sample for XRD was supported on a glass substrate.

## Results and Discussion

In a typical polyol process, synthesis of Ag nanocubes involves the reduction of AgNO_3_ by EG as following reactions^28^:


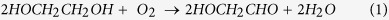



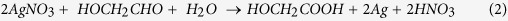


The actual reducing agent in the reduction reaction is glycolaldehyde, which results from the oxidation of EG in oxygen atmosphere at 160 °C. The obtained Ag atoms will start to nucleate and grow into clusters, seeds and then nanocrystals. The initial nucleation process plays an important role in determining the shape of final products. In order to acquire desired nanostructure with good uniformity, the nucleation process should be precisely controlled. It is well known that Ag nanocrystals come from different Ag crystal seeds which exist in the nucleation process. Typically, Ag nanocubes are believed to originate from single-crystal seeds while Ag right bipyramids are supposed to arise from single twinned seeds^27^,^29^, and the multiple twinned seeds are expected to produce other nanostructures such as Ag nanowires^30^. As multiple twinned and single twinned seeds have more defects and lower surface energy compared with single-crystal seeds, the twinned seeds are more favorable for the deposit of reduced Ag atoms. Thus, to obtain Ag nanocubes, both single twinned and multiple twinned seeds should be effectively eliminated. Control over the seeds in the early nucleation stage is essential for the geometry and crystallinity of final nanostructures.

**Figure 1:**
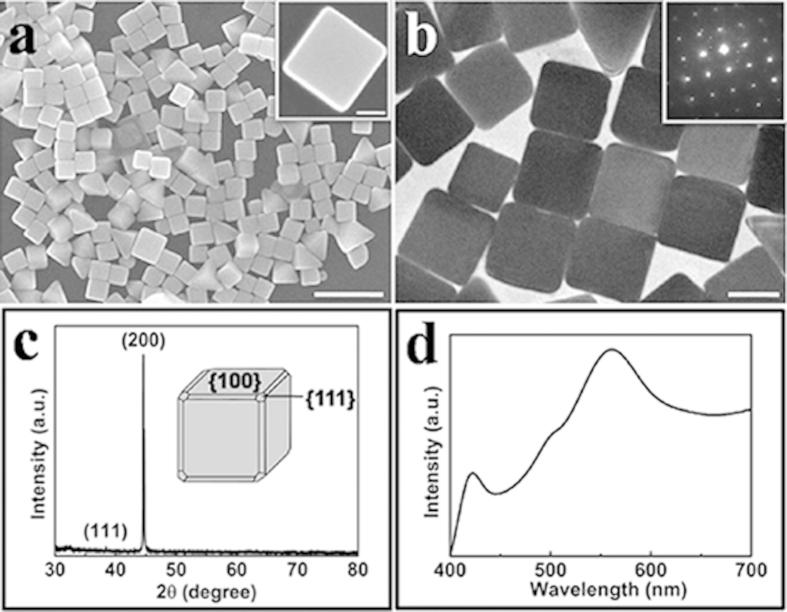
(a) SEM images of a typical sample of Ag nanocubes taken at 90 min, and the inset shows the high-magnification SEM image of a single Ag nanocube. The scale bar is 500 nm and 50 nm respectively. (**b**) TEM image of the same sample. The inset shows the electron diffraction pattern obtained by directing the electron beam perpendicular to one of the square faces of a Ag nanocube. The scale bar is 100 nm. (**c**) XRD pattern of the same batch of Ag nanocubes. (**d**) The extinction spectrum of the Ag nanocubes.

As illustrated in Fig. 1a, Ag nanocubes with large quantities and good uniformity are achieved through improved oxidative etching effect of Br^−^ ions in the polyol synthesis. The mean edge length of Ag nanocubes is 130 nm, with a standard deviation of 10 nm, as demonstrated in the grain diameter distribution map (see supplementary information Fig. S1). It can be clearly observed that there are small quantities of Ag right bipyramids exist which cannot be completely avoided in a synthesis. Ag nanocubes dominate in the products at a percentage of 79%. This is a high value compared with previous reports using Br^−^ ions as addictive ions, where the proportion of Ag nanocubes is less than 20%^27^,^31^. The surfaces of Ag nanocubes are smooth with very few observed defects while the corners are slightly truncated, which can be seen obviously in TEM images (Fig. 1b). The inset shows the electron diffraction pattern obtained by aligning the electron beam perpendicular to one of the square faces of a Ag nanocube. The pattern indicates that obtained nanocubes are single-crystal bounded mainly by {100} facets. The XRD pattern recorded from the same batch of sample is displayed in Fig. 1c. Two peaks can be assigned to the (111), (200) planes of pure fcc Ag respectively. The huge intensity difference between these two peaks is probably due to the situation that the sample is exclusively comprised of nanocubes that are preferentially oriented with their {100} planes parallel to the supporting substrate. Figure 1d shows the optical extinction spectrum of Ag nanocubes. The peaks located at 560 nm and 420 nm may be induced by the dipole resonance and quadrupole resonance of Ag nanocubes respectively, which are consistent with previous report^32^. The prepared Ag nanocubes are good candidates for SERS measurements (see supplementary information Fig. S3).

**Figure 2:**
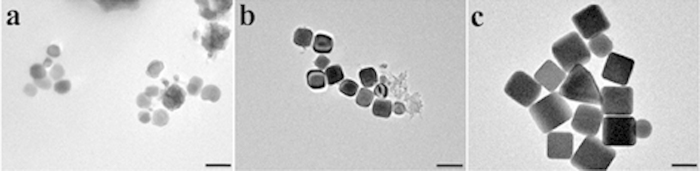
TEM images of samples taken at different stages of a typical synthesis. (**a**) t = 15 min, (**b**) t = 30 min,(**c**) t = 60 min. The reaction temperature is 160 °C. The scale bar is 100 nm.

In order to know the detail of growth process, Ag nanocubes taken at different stages of a synthesis are obtained and investigated by TEM, as illustrated in Fig. 2. It can be observed that there are irregular nanoparticles in Fig. 2a, which are taken after the reaction proceeding for 15 minutes. The light yellow color of the solution (see supplementary information Fig. S2), together with Energy Dispersive X-ray spectroscopy (EDX) spectra, indicate that Ag nanocrystals are formed during this period. Although twinned and single-crystal Ag seeds coexist in the solution in the early stage, twinned nanoparticles are the most abundant nanostructure due to the fact that their surface energies are relatively lower than that of single-crystal^23^. With the existence of Br^−^ ions which are supposed to have the oxidative etching effect on twinned crystal seeds^27^,^29^, most twinned crystal seeds are eliminated with the reaction proceeding. As a result, single-crystal seeds are the primary seeds. The reduced Ag atoms continuously deposit on these crystal seeds. For Ag crystals, the surface free energies (γ) associated with different facets increase in the following order^33^: γ {111} < γ {100} < γ {110}. Therefore, {100} facets are supposed to have a relatively higher growth rate than {111} facets. It is well known that PVP can selectively bind to Ag (100) surfaces^34^,^35^, which thus largely decrease the growth rate of {100} planes. The reduced Ag atoms then deposit on the {111} planes of existing single-crystal Ag seeds, resulting in the formation of Ag nanocubes enclosed preferentially by {100} facets. As shown in Fig. 2b, small nanocubes with relative uniform size are formed. The edge size is about 60 nm, with a standard deviation of 10 nm. With the reaction proceeding, the size of these nanocubes will be increased by absorbing the reduced Ag atoms while the shape is kept the same. It can be seen from Fig. 2c that large Ag nanocubes with size of 115 nm are obtained after 60 minutes. There are a few Ag right bipyramids with edge size of 130 nm in the final products. This is probably due to that single twinned crystal seeds in the initial stage cannot be completely eliminated and the residual single twinned crystals can continuously absorb Ag atoms and grow into Ag right bipyramids.

The growth process of Ag nanocubes demonstrates that oxidative etching effect of Br^−^ ions can be improved to a level that both multiple twinned and single twinned seeds can be effectively removed. The enhancement of oxidative etching has been achieved by increasing the concentration of Br^−^ ions. The ratio between Br and Ag is 7.66 × 10^−3^ in our work, which is six times higher than that in previous reports^27^ (see supplementary information Table S1). Therefore, the main products are Ag nanocubes in our experiments while Ag right bipyramids are primary products in their work. Hence, by the change of concentration of Br^−^ ions, we can selectively remove the crystal seeds and tune the shape of final products in a large extent.

**Figure 3:**
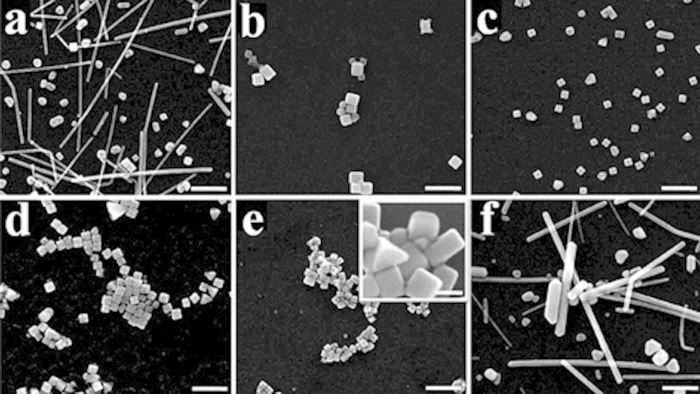
A series of SEM images of the geometry of Ag nanostructures synthesized with different concentrations of NaBr in the previous EG solution. (**a**) 0 mM, (**b**) 0.044 mM, (**c**) 0.094 mM, (**d**) 0.144 mM, (**e**) 0.194 mM, (**f**) 0.244 mM. All the samples are taken at t = 60 min, and the reaction temperature is 160 °C. The scale bar is 500 nm. The scale bar of inset in Fig. 3e is 100 nm.

The influence of the concentration of Br^−^ ions on the geometry of Ag nanostructures is further investigated in our experiments, as shown in Fig. 3. These serial experiments are under same reaction conditions except that the concentration of Br^−^ ions in the previous EG solution (before being injected into the flask) is increased from 0.044 mM (Fig. 3b) to 0.244 mM (Fig. 3f), with Fig. 3a denoting the results of no Br^−^ ions adopted in the previous EG solution. It is found that the ideal concentration of Br^−^ ions to obtain Ag nanocubes with good uniformity and large quantities is 0.144 mM in these serial experiments. Ag nanowires and irregular Ag nanoparticles are the primary products when Br^−^ ions are excluded in the previous EG solution, owing to the fact that neither multiple twinned seeds nor single twinned seeds can be effectively removed from the initial stage. When 0.044 mM Br^−^ ions are adopted as Fig. 3b shown, a large amount of rectangular nanoparticles as well as some nanocubes exist. It results from that both multiple twinned and single twinned seeds have been eliminated by Br^−^ ions, which indicates the oxidative etching effect of Br^−^ ions has been enhanced by increasing the concentration of Br^−^ ions. However, the amount of Br^−^ ions at this concentration is not enough to maintain the isotropic growth of single-crystal seeds, leading to the rectangular nanoparticles rather than nanocubes dominate in the final products. With the increase of the concentration of Br^−^ ions, the isotropic growth of single-crystal seeds can be well kept. Hence, Ag nanocubes become the major component in the final products, illustrated by Fig. 3c and Fig. 3d. The edge size of nanocubes is 85 nm in Fig. 3c, smaller than that in Fig. 3d. We ascribe this phenomenon to the increased growth rate of nanocubes. In detail, the increase of the concentration of Br^−^ ions can enhance the release rate of Ag atoms from multiple twinned and single twinned seeds, which can improve the deposition rate of Ag atoms on the single-crystal seeds. In addition, the uniformity and quantity of nanocubes in Fig. 3d are also improved. This good situation, however, cannot be maintained when further increasing the concentration of Br^−^ ions. As shown in Fig. 3e, the size of nanocubes becomes small and some irregular nanoparticles start to appear. This is probably due to that the increased introduction of Br^−^ ions into the solution will improve the probability of the combination of Br^−^ and Ag^+^, and thus the influence of AgBr cannot be ignored any more. The formation of AgBr can reduce the amount of free Ag^+^ in the initial stage and prevent the solution from being rapidly supersaturated with Ag seeds. As a result, nanocubes with small size are formed due to the decreased deposition rate of Ag atoms. Meanwhile, a small amount of AgBr, which probably serve as sites for the growth of twinned seeds like the function of AgCl^23^, can account for the appearance of irregular nanoparticles in Fig. 3e. When the concentration of Br^−^ ions is still increased, a large amount of nanowires and irregular nanoparticles can be observed in Fig. 3f.

These experimental results provide strong evidence that Br^−^ ions can not only etch away multiple twinned seeds, but also the single twinned seeds. Whether single twinned seeds can be effectively eliminated or not depends on the concentration of Br^−^ ions used in the experiment. With the increase of the concentration of Br^−^ ions, the oxidative etching effect can be enhanced. In order to obtain a large quantity of Ag nanocubes, the concentration of Br^−^ ions should be kept at a suitable range. It can be seen from Fig. 3 that the shape of Ag nanostructures is very sensitive to the concentration of Br^−^ ions, and the increase of 0.05 mM in the concentration of Br^−^ ions will bring a large influence on the size and shape of final products. Therefore, this work provides us with an effective way to control the size and shape of Ag nanostructures through the change of the concentration of Br^−^ ions.

The ratio of Br to Ag in each case (Fig. 3) is calculated and presented in Table 1. The optimal concentration range of Br^−^ ions for the high-quality synthesis of Ag nanocubes in our experiments is from 0.094 mM to 0.194 mM, with the molar ratio of Br to Ag being 5.00 × 10^−3^, 7.66 × 10^−3^, and 10.32 × 10^−3^ correspondingly, which are all larger than that in the previous reports of synthesis of Ag right bipyramids^27^ (see supplementary information Table S1). This comparison data further provides evidence to support our proposed understanding of the enhanced oxidative etching based on the increased concentration of Br^−^ ions. Moreover, when the molar ratio is decreased to 2.34 × 10^−3^, most of the final products are rectangular nanoparticles as shown in Fig. 3b, which is consistent with the reports of the synthesis of Ag nanobars^31^ (see supplementary information Table S1). It should be noted that the follow-up experiment is carrying on, that through precisely controlling the molar ratio of Br to Ag to realize the continual geometry change from Ag right bipyramids to nanocubes and other nanostructures.

**Figure 4:**
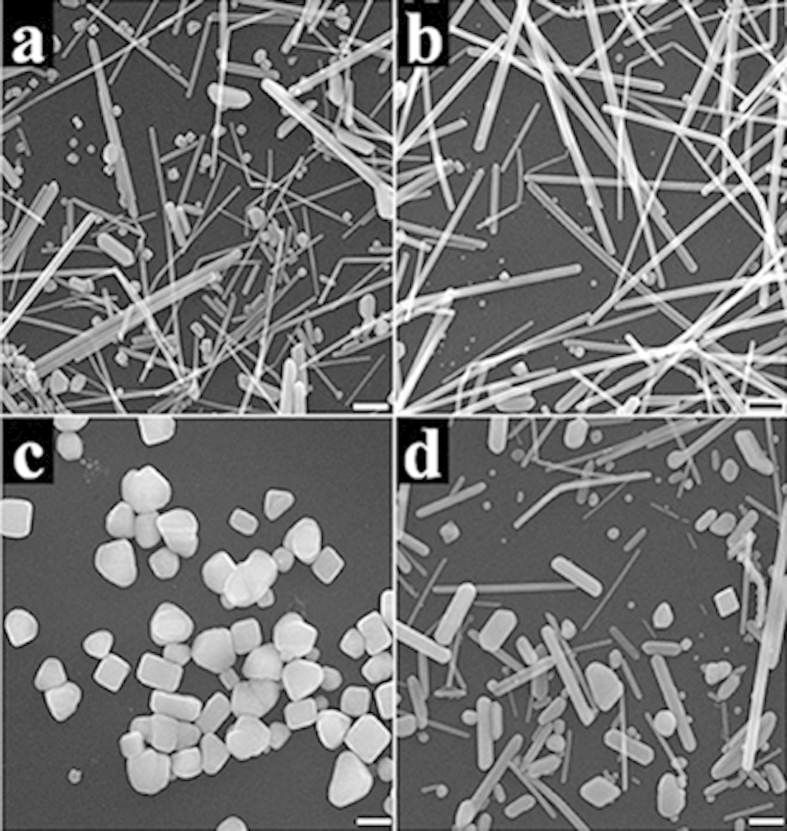
SEM images of the final products obtained when the ratio of PVP to AgNO_3_ is changed to (a) 1:1, (b) 2:1, and the reaction temperature is altered to (c) 140 °C, (d) 180 °C while other conditions are kept the same as previous standard synthesis conditions. The scale bar is 500 nm.

The influence of reaction temperature and molar ratio of PVP to AgNO_3_ on the synthesis of Ag nanocubes have also been investigated. It is proposed that PVP can change the growth rate of Ag {100} planes, and can work as capping agent to prevent the nanostructures from aggregation^29^,^36^. The suitable molar ratio of PVP to AgNO_3_ is 1.5:1 in our work. Either increasing or decreasing the amount of PVP can induce the appearance of irregular nanoparticles and nanowires, as shown in Fig. 4a and Fig. 4b. The change of reaction temperature can also affect the shape of final products, which can be clearly observed in Fig. 4c and Fig. 4d. The temperature mainly influences the reaction rates, which is important for the growth process. Therefore, reaction conditions such as temperature and ratio of PVP to AgNO_3_ in the system should be kept at suitable values to maintain the uniformity of Ag nanocubes.

## Conclusions

We have synthesized large quantities of Ag nanocubes with good morphology by employing Br^−^ ions. With systematically studying the effect of Br^−^ ions, we found that oxidative etching effect can be largely enhanced by increasing the concentration of Br^−^ ions and the geometry of nanostructure is sensitive to the concentration of Br^−^ ions. Consequently, geometry of Ag nanostructure can be controlled through manipulating the concentration of Br^−^ ions. Our findings provide an effective way to control the shape of metallic nanostructures.

## Additional Information

**How to cite this article**: Wu, F. *et al*. Bromide (Br) - Based Synthesis of Ag Nanocubes with High-Yield. *Sci. Rep*. **5**, 10772; doi: 10.1038/srep10772 (2015).

## Supplementary Material

Supplementary Information

## Figures and Tables

**Table 1:** The molar ratio of Br to Ag at different concentrations of Br^−^ ions adopted in Fig. 3.

Amount of substance (×10^−3^ mol)	3a 0 mM	3b 0.044 mM	3c 0.094 mM	3d 0.144 mM	3e 0.194 mM	3f 0.244 mM
**Br**	0	5.94 × 10^−3^	12.69 × 10^−3^	19.44 × 10^−3^	26.19 × 10^−3^	32.94 × 10^−3^
**Ag**	2.538	2.538	2.538	2.538	2.538	2.538
**Br: Ag**	0	2.34	5.00	7.66	10.32	12.98
