# Supplementary material for: Bromide (Br) - Based Synthesis of Ag Nanocubes with High-Yield
Source: Sci Rep. 2015 Jun 9;5:10772. doi: 10.1038/srep10772 (PMC4460816; doi:10.1038/srep10772)
Supplement: Supplementary Information [file srep10772-s1.pdf]

Bromide (Br) - Based Synthesis of Ag Nanocubes with High-Yield

Fan Wu, Wenhui Wang*, Zhongfeng Xu, Fuli Li

School of Science, Xi'an Jiaotong University, Xi'an 710049, China

* Correspondence
E-mail: w.wang@mail.xjtu.edu.cn, Tel.: 029-82663394

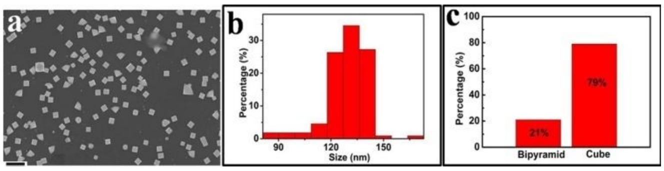

Figure S1. (a) SEM images of the same sample of Ag nanocubes, the scale bar is 500 nm; (b) the grain diameter distribution map of Ag nanocubes. These Ag nanocubes have a mean edge length of 130 nm, with a standard deviation of 10 nm; (c) the shape distributions of Ag nanocrystals. The nanocubes account for 79 percent of the number of products, and the bipyramids are delivering up to 21 percent of the products.

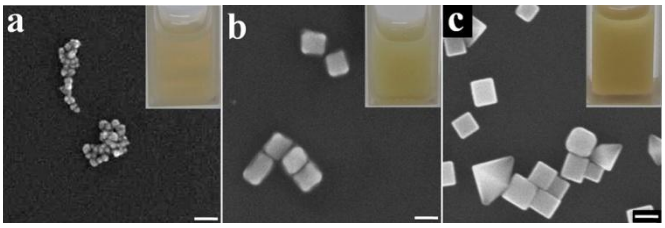

Figure S2. SEM images of samples taken at different stages of synthesis. (a) t = 15 min, (b) t = 30 min, (c) t = 60 min. The reaction temperature is 160 ℃. The scale bar is 100 nm. All the insets are corresponded to the colors of solutions taken at different time.

**Amount of substance ($\times 10^{-3}$ mol)**	**bipyramid**	**nanobar**	**cube**
**Br**	0.33×10^{-3}	0.66×10^{-3}	19.44×10^{-3}
**Ag**	282×10^{-3}	282.57×10^{-3}	2538×10^{-3}
**Br : Ag**	1.17	2.34	7.66

Table S1. The molar ratio of Br to Ag reported by Xia's group[27,31], in addition, the last column is the results in our experiments.

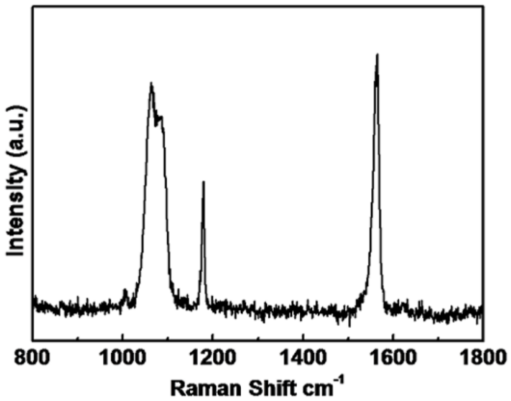

Figure S3. SERS spectrum of 1, 4-BDT on a single Ag nanocube from the same sample. λ = 633 nm, P = 0.5 mW, t = 10 s. The peaks at 1180 cm^{-1} and 1564 cm^{-1} can be assigned to the 9a vibrational mode (CH bending) and 8a vibrational mode (phenyl ring stretching motion) respectively.
